# Case Report: From *Talaromyces marneffei* infection to EBV-positive lymphoma in an adult with anti-IFN-γ autoantibody-associated immunodeficiency

**DOI:** 10.3389/fimmu.2026.1783319

**Published:** 2026-04-21

**Authors:** Yan Ning, Xuemei Huang, Hanlin Liang, Siqiao Liang, Jiaqi Qin, Ting Chen, Shilian Liang, Xiaona Liang, Feixiang Tan, Ni Chen, Siyao Wu, Limei Hong, Zengtao Luo, Zhiyi He

**Affiliations:** 1 Department of Respiratory and Critical Care Medicine, The First Affiliated Hospital of Guangxi Medical University, Nanning, Guangxi, China; 2 Wuming Hospital of Guangxi Medical University, Nanning, Guangxi, China; 3 Department of Respiratory and Critical Care Medicine, Qinzhou Second People ‘s Hospital, Qinzhou, Guangxi, China; 4 Department of Respiratory and Critical Care Medicine, Wuzhou Worker’s Hospital, Wuzhou, Guangxi, China

**Keywords:** adult-onset immunodeficiency, anti-interferon-γ autoantibodies, CREBBP mutation, Epstein–Barr virus-positive lymphoma, *Talaromyces marneffei*

## Abstract

**Background:**

Anti–interferon-γ autoantibodies (AIGAs) are an established cause of adult-onset immunodeficiency (AOID), predisposing individuals to disseminated intracellular infections such as *Talaromyces marneffei* (TM). However, their role in promoting persistent immune dysregulation and subsequent lymphomagenesis remains poorly understood.

**Case presentation:**

A 63-year-old Chinese female with high-titer AIGAs (1:2500) initially presented with disseminated TM. Despite antifungal therapy, her clinical course was complicated by recurrent opportunistic infections—including *Varicella-zoster virus* (VZV) and *Mycobacterium persicum* (*M. persicum*), and steroid-dependent inflammatory episodes. Approximately two years after the initial presentation, she developed an *Epstein-Barr virus* (EBV)-positive aggressive B-cell lymphoma, confirmed by mass biopsy and PET/CT. Despite treatment with rituximab and broad-spectrum antimicrobials, she died of Gram-negative septic shock. Whole-exome sequencing (WES) revealed a CREBBP p.Q278P mutation, providing a genetic perspective on her disease susceptibility.

**Conclusion:**

This case illustrates a rare progression from AIGAs-associated immunodeficiency to EBV-driven lymphoma, suggesting a “triple-hit” pathogenic model that warrants further investigation, comprising: (1) AIGAs-associated AOID; (2) chronic antigenic stimulation from persistent infections that may exacerbate immune dysregulation; and (3) a CREBBP mutation that may act as a genetic contributor to malignant transformation. This case underscores the necessity for rigorous tumor surveillance and individualized treatment in patients with AIGAs-associated immunodeficiency.

## Introduction

Anti-interferon-γ autoantibodies (AIGAs) associated adult-onset immunodeficiency (AOID) is a rare acquired immune disorder characterized by the neutralization of interferon-γ (IFN-γ), which significantly increases susceptibility to intracellular pathogens such as *Talaromyces marneffei* (TM) and *non-tuberculous mycobacteria* (NTM) (1–3). This syndrome has a complex clinical presentation, featuring recurrent disseminated opportunistic infections as well as autoimmune manifestations and chronic inflammation (3, 4). Recent evidence has linked AIGAs-associated AOID to a markedly heightened risk of malignancies—especially *Epstein-Barr virus* (EBV)-associated lymphomas—implicating immune dysregulation as a key mediator of the “infection-inflammation-cancer” pathological cascade (5, 6).

Disseminated TM infection is a hallmark of AIGAs-associated AOID, often following a chronic and relapsing course. Despite standard antifungal therapy, the infection tends to progress, disseminating to multiple sites including the skin and bones. Furthermore, the impaired IFN-γ signaling pathway compromises immune surveillance against latent viruses like EBV, thereby increasing the risk of EBV reactivation and subsequent EBV-associated B-cell lymphomagenesis (7, 8). While several cohort studies have reported an association between AIGAs and neoplasms, the precise underlying mechanisms and potential genetic predispositions remain poorly understood (5).

Herein, we report a comprehensive case of an AIGAs-associated disseminated TM patient who subsequently developed aggressive EBV-positive B-cell lymphoma. Integrating Whole-exome sequencing (WES) data that identified a CREBBP gene mutation, we explore its potential pathogenic role in the context of immunodeficiency and lymphomagenesis. Through this case, we aim to elucidate the interplay mechanisms underlying the “chronic infection–immune dysregulation–tumorigenesis” axis in AIGAs-associated AOID and provide evidence-based insights for the clinical management and long-term surveillance of such patients.

## Case presentation

A 63-year-old immunocompetent woman presented to our tertiary care center in April 2024 with an 8-month history of persistent fever and respiratory symptoms. Her illness had commenced in August 2023 with high-grade fever (38-39 °C), productive cough with tenacious white sputum, and characteristic sharp, stabbing pain in the thoracolumbar region. Initial diagnostic workup at a referring institution utilizing metagenomic next-generation sequencing (mNGS) of bronchoalveolar lavage fluid (BALF) confirmed TM infection. The patient demonstrated initial clinical response to sequential antifungal therapy with amphotericin B followed by voriconazole, and was transitioned to maintenance itraconazole. However, persistent constitutional and respiratory symptoms accompanied by severe cephalgia necessitated referral to our facility for further management. Her medical history was significant for neurovascular headaches. Physical examination upon admission documented an afebrile state with stable hemodynamic parameters. Cardiopulmonary assessment revealed scattered wet rales bilaterally without evidence of consolidation. Initial laboratory investigation demonstrated leukocytosis (11.13 × 10^9^/L) with neutrophilic predominance (71.8%), accompanied by mild anemia (Hb 110 g/L) and relative lymphopenia (18.2%). Markers of systemic inflammation were substantially elevated, including C-reactive protein (CRP 64.89 mg/L), erythrocyte sedimentation rate (ESR 75 mm/h), and procalcitonin (0.127 ng/mL). Immunological profiling revealed elevated immunoglobulin levels, with serum IgE measuring 152.0 IU/mL and IgG elevated to 23.06 g/L. Critical diagnostic testing revealed high-titer AIGAs (1:2500) by enzyme-linked immunosorbent assay (ELISA), and their neutralizing capacity was subsequently confirmed by Western blotting. Thoracic computed tomography demonstrated extensive lymphadenopathy involving mediastinal, hilar, and bilateral axillary stations. Whole-body scintigraphy revealed multifocal skeletal involvement with increased metabolic activity, consistent with disseminated mycotic disease.

**Figure 1:**
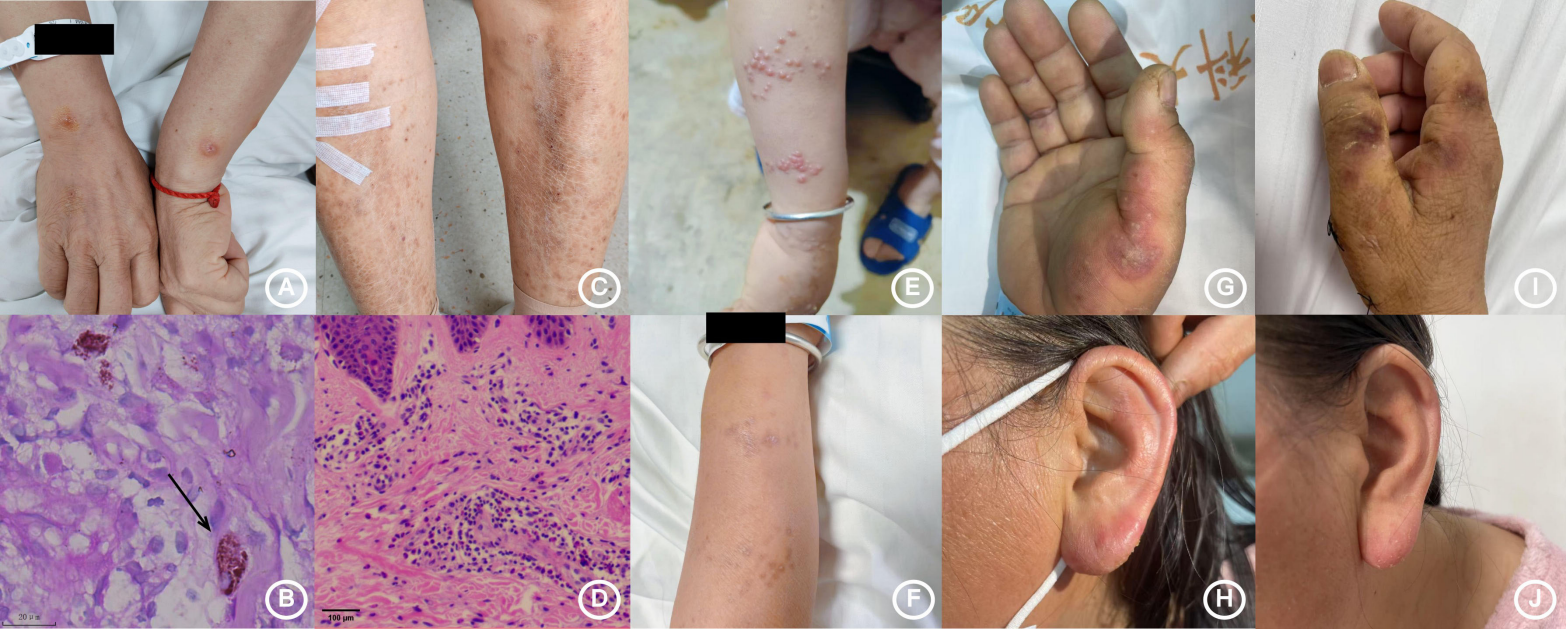
Cutaneous manifestations and histopathological findings during the disease course. **(A, B)** Disseminated *Talaromyces marneffei* infection. **(A)** Cutaneous presentation as symmetrical vesiculopustular eruptions on the volar wrist. **(B)** Histopathological findings of disseminated *Talaromyces marneffei* infection. Skin biopsy specimen showing intracellular yeast-like fungi (Periodic acid–Schiff stain, ×80). Arrows indicate the fungal organisms. **(C, D)** Lesions consistent with eosinophilic dermatitis. **(E, F)** *Varicella-zoster virus* infection: acute vesicular stage **(E)** and post-treatment resolution **(F)**. **(G, H)** Cutaneous manifestations suspected to be associated with *Mycobacterium persicum* infection. **(I, J)** Appearance of skin lesions following combination antimicrobial therapy.

The therapeutic course was complicated by recurrent febrile episodes and persistent respiratory symptoms despite appropriate antifungal coverage. Empirical antimicrobial therapy was expanded to include moxifloxacin for suspected nontuberculous mycobacterial co-infection while maintaining itraconazole prophylaxis. During hospitalization, the patient developed a distinctive cutaneous manifestation characterized by pruritic vesiculopustular eruptions distributed symmetrically on the volar wrists and anterior tibial surfaces (**Figure 1A**). Histopathological examination of skin biopsy specimens confirmed ongoing TM dissemination (**Figure 1B**). In the context of persistent systemic inflammation evidenced by elevated acute-phase reactants and hypergammaglobulinemia, adjunctive corticosteroid therapy with prednisone (25 mg daily) was initiated to modulate the excessive immune activation. This intervention resulted in satisfactory clinical improvement, permitting hospital discharge.

**Figure 2:**
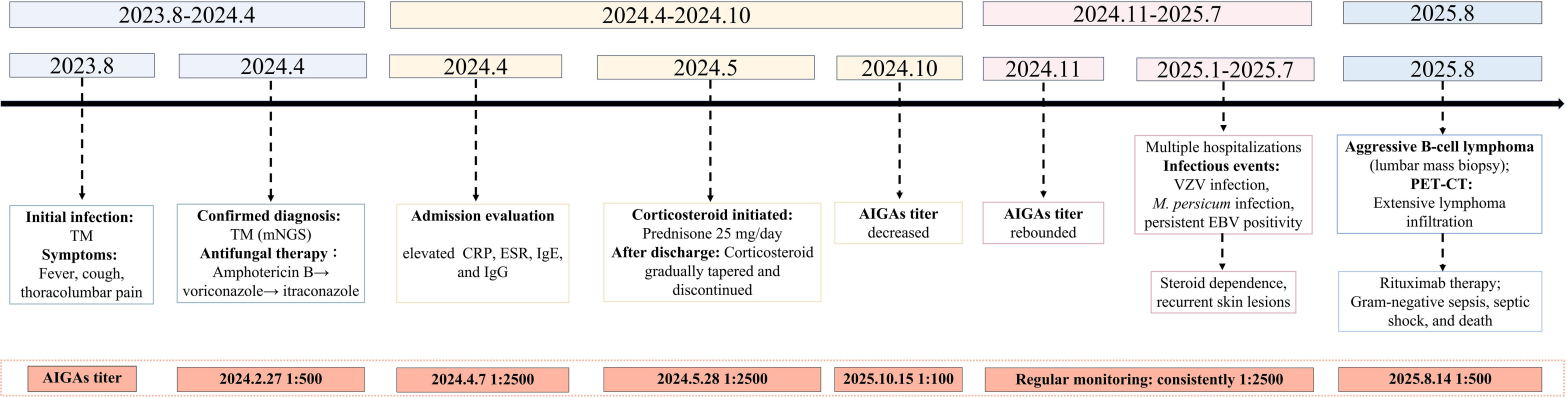
Dynamic changes in AIGAs titers aligned with clinical events during the disease course.

The post-discharge therapeutic regimen comprised combination therapy with itraconazole, azithromycin, prednisone acetate, and moxifloxacin. Through careful outpatient monitoring, the corticosteroid component was successfully tapered and discontinued over a two-month period. Early September 2024 witnessed the emergence of gastrointestinal intolerance manifesting as nausea and anorexia, attributed to polypharmacy effects. Subsequent evaluation confirming disease stability prompted cessation of all anti-infective agents. Longitudinal serological monitoring demonstrated dynamic fluctuation in anti-interferon-γ autoantibody titers, declining to 1:100 by October 2024 before rebounding to the initial high titer (1:2500) in November 2024 (**Figure 2**). The period spanning January to July 2025 was characterized by recurrent hospitalizations for diverse and refractory dermatological manifestations, reflecting the progressive nature of her underlying immune dysregulation (**Figures 1C–J**, **Table 1**).

**Table 1:** Summary of major infectious events and clinical course.

Time point	Major events / Symptoms	Pathogen	Diagnostic method	Treatment
Aug-23	Fever, cough, thoracolumbar stabbing pain	TM	mNGS (bronchoalveolar lavage fluid)	Amphotericin B + voriconazole, then switched to itraconazole maintenance
Apr 2024 (admission)	Persistent fever, respiratory symptoms, generalized lymphadenopathy, multifocal skeletal involvement	TM (disseminated)	Skin biopsy (PAS stain)	Itraconazole + moxifloxacin (empiric anti-NTM), plus prednisone for immune modulation
Oct 2024 –Jul 2025	Recurrent dermatological manifestations (various morphologies)	Eosinophilic dermatitis, VZV, *M. persicum*, etc.	Skin biopsy, mNGS	Pathogen-directed anti-infective therapy combined with immunomodulation
Jul-25	Lumbar pain, abdominal distension, anorexia, pancreatitis	EBV-positive B-cell lymphoma	Lumbar mass biopsy (histopathology + EBER in situ hybridization), mNGS , PET/CT	Rituximab (discontinued due to infection)
Aug-25	Gram-negative sepsis, septic shock	Gram-negative bacteria (species not specified)	Blood culture, clinical assessment	Liposomal amphotericin B, meropenem, combination antibacterial therapy; patient died of refractory septic shock

**Figure 3:**
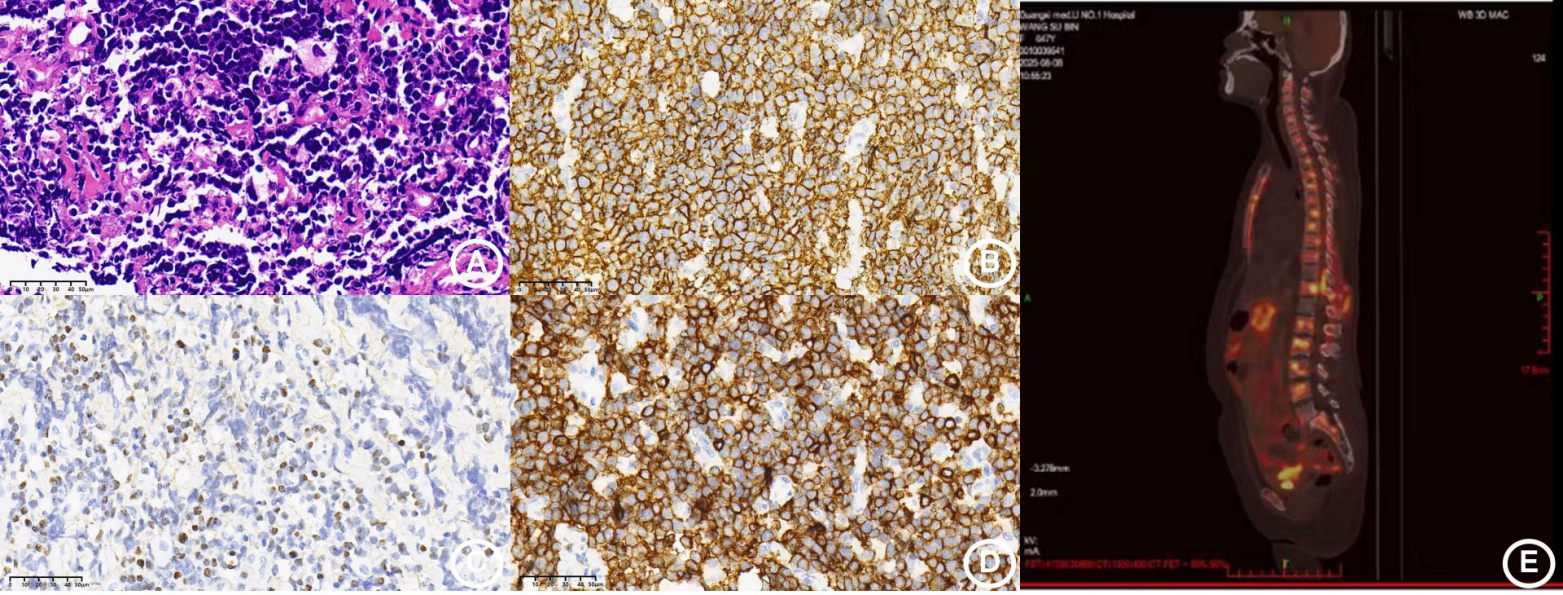
Pathological, immunohistochemical, and PET-CT imaging features of the lymphoma. **(A)** Diffuse proliferation of lymphocytes characterized by oval nuclei, inconspicuous nucleoli, and scattered mitotic figures (H&E stain, ×40). **(B)** Immunohistochemistry reveals diffuse positivity for CD20 (×40). **(C)** *In situ* hybridization demonstrates positivity for Epstein–Barr virus-encoded small RNAs (EBERs) (×40). **(D)** Immunohistochemistry reveals diffuse positivity for CD79a (×40). **(E)** PET/CT scan shows extensive hypermetabolic lesions consistent with disseminated lymphomatous involvement.

The subsequent clinical deterioration was marked by the development of lumbar pain, abdominal distension, and anorexia, accompanied by elevated pancreatic enzymes (amylase 598 U/L, lipase 887.8 U/L). Abdominal imaging confirmed pancreatitis with associated retroperitoneal involvement. Histopathological evaluation of a lumbar mass biopsy demonstrated EBV-positive aggressive B-cell non-Hodgkin lymphoma (**Figures 3A–D**), with mNGS confirming high viral load (3,856 sequences). PET-CT imaging revealed extensive hypermetabolic activity consistent with disseminated lymphomatous involvement (**Figure 3E**). The therapeutic regimen was complicated by the emergence of Gram-negative sepsis during immunochemotherapy with rituximab. Despite intensive antimicrobial therapy including liposomal amphotericin B, meropenem, and combination antibacterial agents, the patient developed refractory septic shock and died in August 2025.

To investigate the potential immunogenetic basis of the patient’s recurrent opportunistic infections and lymphoma development, we performed WES on peripheral blood. This identified a missense mutation in the CREBBP gene (c.A833C: p.Q278P). Despite its “benign” annotation in population databases, the CREBBP gene encodes a crucial tumor suppressor and epigenetic regulator central to lymphocyte development, activation, and immune surveillance. Given the patient’s severe clinical phenotype, this variant is postulated to have contributed to both the immunodeficiency and lymphomagenesis.

## Discussion

This case comprehensively illustrates the rare progression from AIGAs-associated disseminated TM to EBV-positive aggressive B-cell lymphoma, and elucidates the “chronic infection–immune dysregulation– tumorigenesis” axis in adult-onset immunodeficiency.

The patient’s initial presentation aligned with the core features of AIGAs-associated AOID, wherein AIGAs neutralize IFN-γ and impair immune defenses against intracellular pathogens and dimorphic fungi (2, 3, 9). This immune dysregulation manifested as both disseminated TM infection and EBV reactivation, highlighting the critical role of impaired cellular immunity (5, 6). Dynamic fluctuations in AIGAs titers (from 1:100 to 1:2500) provided direct evidence of persistent immune dysregulation. Impaired IFN-γ signaling led to refractory infections; despite standard antifungal therapy, the patient experienced recurrent progression of skin and bone lesions. Notably, the rise in AIGAs titer preceded the diagnosis of bone lesions and lymphoma, suggesting its potential as a biomarker for disease activity. This finding is supported by Tsai et al., who reported that patients with persistently high or rising antibody titers have a significantly increased risk of delayed malignancies (5). Mechanistically, AIGAs block IFN-γ signaling, impairing the anti-tumor activity of CD8+ T cells and NK cells, thereby creating a microenvironment conducive to immune escape and EBV-associated malignancies (10, 11).

EBV establishes latent infection in B lymphocytes in approximately 90% of healthy adults, and its reactivation is closely linked to B-cell malignant transformation under immunodeficient conditions (12). In the present case, EBV was detected by mNGS in both BALF and a lumbar mass biopsy (sequence count: 3,856). Pathological examination confirmed EBV-positive aggressive non-Hodgkin B-cell lymphoma, suggesting a potential etiological link between EBV infection and lymphomagenesis. The underlying mechanisms may involve a dual-driver process. First, AIGAs-associated immunodeficiency likely creates an immune-permissive microenvironment that facilitates EBV reactivation and impairs cytotoxic T-cell clearance of EBV-infected B cells (6, 13, 14). Second, persistent TM and probable nontuberculous mycobacterial infections sustain chronic inflammation, as evidenced by elevated CRP, ESR, and immunoglobulin levels, which may drive upregulation of pro-inflammatory cytokines such as IL-6 and tumor necrosis factor-α, thereby potentially accelerating proliferation and malignant transformation of EBV-infected B cells (15). However, given the single-case nature of this observation, causal relationships cannot be established, and the proposed mechanisms remain speculative.

In addition to the classic EBV-associated lymphomagenesis pathway, chronic active Epstein–Barr virus disease (CAEBVD) represents another EBV-driven entity that may contribute to the clinical course in immunodeficient patients (16). CAEBVD is characterized by persistent or recurrent infectious mononucleosis-like symptoms and progressive lymphoproliferation, often culminating in lymphoma (16). Although the patient did not meet the classical diagnostic criteria for CAEBVD—including prolonged fever, lymphadenopathy, and hepatosplenomegaly lasting more than three months with elevated EBV DNA loads—the sustained immune dysregulation secondary to AIGAs may have created a permissive environment for chronic EBV reactivation, potentially overlapping with features of CAEBVD (15, 17, 18). Further investigation into the continuum between EBV reactivation, CAEBVD, and overt lymphomagenesis in the context of AIGAs-associated AOID is warranted.

Although the chronic infection–immunodeficiency–malignancy axis is well established in HIV infection and primary immunodeficiencies, emerging evidence suggests it may also occur in AOID. Tsai et al. reported a cancer incidence of up to 25% in an AIGA cohort, noting that chronic infection combined with autoimmune-mediated persistent inflammation creates a soil conducive to tumorigenesis (5). The present case supports this concept and highlights the need for long-term EBV viral load and tumor marker monitoring in AIGAs-positive patients with chronic infections to facilitate early detection.

WES identified a *CREBBP* p.Q278P mutation, providing a genetic perspective on lymphomagenesis. *CREBBP* encodes a transcriptional coactivator with histone acetyltransferase activity and serves as a master regulator of T-cell-dependent immune responses and germinal center B-cell reactions (19, 20). Although rare in public databases and annotated as benign, this mutation may act as a hypomorphic allele in complex diseases, compromising immune stability. Its enabling role is twofold: first, CREBBP insufficiency exacerbates AIGAs-associated T-cell immunodeficiency, thereby weakening immune surveillance of EBV-infected cells and facilitating EBV reactivation and B-cell proliferation (21); second, as a tumor suppressor, CREBBP is frequently inactivated in follicular lymphoma and germinal center-derived diffuse large B-cell lymphoma (22), and its loss of function disrupts histone acetylation and p53 signaling, driving genomic instability and lymphomagenesis (21). Based on these findings, we propose a “three-hit” hypothesis as a potential mechanistic framework for understanding the pathogenesis in this case: the first hit is acquired immunodeficiency resulting from high-titer AIGAs neutralizing IFN-γ, increasing susceptibility to intracellular pathogens; the second hit involves persistent antigenic stimulation from chronic TM, NTM and EBV infections, triggering immune activation and dysregulation; the third hit is the CREBBP mutation acting as a genetic vulnerability that may synergize with EBV oncogenesis, potentially contributing to the development of an aggressive B-cell lymphoma clone. While this hypothesis provides a coherent framework, it remains speculative and requires validation in larger cohorts and functional studies.

The patient’s two-year course highlights diagnostic challenges, as overlapping infectious and neoplastic symptoms often lead to misdiagnosis of lymphoma as disseminated infection. We recommend prompt pathological biopsy and PET-CT for AIGAs-positive patients unresponsive to standard anti-infective therapy (23). NGS was crucial for diagnosing TM and EBV in this case, consistent with IDSA guidelines for refractory infections (24, 25).

Lymphoma treatment poses challenges in immunodeficient patients: rituximab, as a foundational therapy for EBV-positive B-cell lymphoma, requires careful risk-benefit assessment in AIGAs-associated AOID with active infections, balancing B-cell depletion-induced immune dysregulation against the heightened risk of worsening infections in an already immunocompromised state (6). This risk was confirmed in the present case by the onset of Gram-negative septic shock 48 hours after rituximab administration, underscoring the necessity of adhering to an “infection control first” principle—initiating low-intensity antitumor therapy only after infection stabilization (26).

AIGAs-associated AOID necessitates individualized comprehensive management. Anti-infective therapy requires adequate-course targeted medications; however, AIGAs-associated immunodeficiency leads to disseminated infections in 92.6% of patients (27), necessitating dynamic monitoring of pathogen load and inflammatory markers to adjust treatment strategies. Immunomodulatory therapy remains exploratory: low-dose IFN-γ may improve symptoms in nontuberculous mycobacterial infections, but its efficacy against TM and lymphoma prevention is unconfirmed (28); AIGA-neutralizing antibody development offers a new direction (9). Glucocorticoids should be reserved for the “high-titer with immune impairment” subtype (54.3%), as studies show that low-dose prednisone reduces AIGAs titers and alleviates symptoms in 67.74% of these patients (29). However, their use should be short-term and accompanied by intensified anti-infective measures. Secondary malignancies should be managed with low-intensity regimens only after infection control, avoiding severe infections induced by immune-depleting agents (26).

## Conclusion

This case illustrates a rare progression from AIGAs-associated AOID to EBV-driven lymphoma, with the CREBBP p.Q278P mutation highlighting a potential genetic susceptibility in this disease cascade. Dynamic AIGAs titers may serve as a biomarker for disease activity and cancer risk stratification. The proposed “triple-hit” hypothesis (AIGAs-mediated immune deficiency, chronic infection-related inflammation, and CREBBP-associated genetic susceptibility) offers a potential framework for understanding disease progression in this case, underscoring the need for long-term EBV and tumor surveillance in AIGAs-positive patients. Further studies are needed to validate this hypothesis and establish causal relationships.

## Data Availability

The original contributions presented in the study are included in the article/supplementary material. Further inquiries can be directed to the corresponding author.
